# Metformin Modulates High Glucose-Incubated Human Umbilical Vein Endothelial Cells Proliferation and Apoptosis Through AMPK/CREB/BDNF Pathway

**DOI:** 10.3389/fphar.2018.01266

**Published:** 2018-11-06

**Authors:** Xiqiong Han, Bilei Wang, Yuning Sun, Jia Huang, Xin Wang, Wenqi Ma, Yi Zhu, Rongfeng Xu, Hong Jin, Naifeng Liu

**Affiliations:** ^1^Department of Cardiology, Zhongda Hospital Affiliated to Southeast University, Nanjing, China; ^2^Department of Cardiology, Shanghai First People’s Hospital Affiliated to Shanghai Jiao Tong University, Shanghai, China

**Keywords:** metformin, diabetes mellitus, HUVECs, AMPK, CREB, BDNF

## Abstract

Cardiovascular disease (CVD) is a leading cause of mortality and morbidity among patients with diabetes. Endothelial dysfunction is an early physiological event in CVD. Metformin, a common oral antihyperglycemic agent, has been demonstrated to directly affect endothelial cell function. Brain-derived neurotrophic factor (BDNF), originally discovered in the brain as a neurotrophin, has also been reported to play a protective role in the cardiovascular system. In our study, we demonstrated that high glucose (HG) reduced cell proliferation and induced cell apoptosis via changes in BDNF expression and that metformin reversed the effects of HG injury by upregulating BDNF expression. Furthermore, we found that cyclic AMP response element binding (CREB) phosphorylation was reduced in HG-treated human umbilical vein endothelial cells (HUVECs), and this effect was reversed by the metformin treatment. However, the metformin effect on BDNF levels in HG-incubated HUVECs was blocked by a CREB inhibitor, which indicated that BDNF expression is regulated by metformin through CREB activation. In addition, we found that adenosine monophosphate-activated protein kinase (AMPK) activation is involved in CREB/BDNF regulation in HG-incubated HUVECs treated with metformin and that an AMPK inhibitor impaired the protective effects of metformin on HG-treated HUVECs. In conclusion, this study demonstrated that metformin affects cell proliferation and apoptosis via the AMPK/CREB/BDNF pathway in HG-incubated HUVECs.

## Introduction

The prevalence of diabetes mellitus, particularly type 2 diabetes mellitus (T2DM), has increased dramatically in recent years, and the number of 20- to 79-year-old patients with diabetes is predicted to increase to 642 million by 2040 (Ogurtsova et al., 2017). Cardiovascular disease (CVD) is a leading cause of mortality and morbidity among patients with diabetes. Approximately 75% of patients with T2DM will die of CVD (Mancini et al., 2017). Chronic hyperglycemia, one of the main etiologies of diabetic complications, is recognized as a major initiator of diabetic vascular complications (De Vriese et al., 2000; Detaille et al., 2005). Studies have indicated that diabetes or high-glucose (HG) levels trigger multiple endothelial dysfunctions, such as dysregulated endothelial cell proliferation, migration, and apoptosis (Baumgartner-Parzer et al., 1995; Tousoulis et al., 2013; Dong et al., 2017). Furthermore, endothelial dysfunction is an early physiological event in CVD (Vanhoutte, 2009).

Metformin is an oral antihyperglycemic agent that is commonly prescribed for treating T2DM. This medication exerts multiple biological effects in addition to its capacity to lower the blood glucose concentration (Viollet et al., 2012). Several clinical trials have suggested that metformin improves vascular function and reduces mortality and cardiovascular end points in patients with T2DM (UK Prospective Diabetes Study (UKPDS) Group, 1998; Roussel et al., 2010) by actions that cannot be ascribed entirely to antihyperglycemic effects (Gundewar et al., 2009). Furthermore, some studies *in vitro* and *in vivo* have demonstrated that metformin directly attenuates endothelial dysfunction. Metformin decreases TNF-α-induced gene expression of proinflammatory and cell adhesion molecules to inhibit endothelial cell inflammation (Hattori et al., 2006) and ameliorates HG-induced endothelial cell death by suppressing mitochondrial permeability transition (Detaille et al., 2005). It also inhibits HG-dependent reactive oxygen species (ROS) overproduction by reducing NADPH oxidase activity in aortic endothelial cells (Batchuluun et al., 2014). In obese diabetic mice, metformin restores endothelial function by inhibiting endoplasmic reticulum (ER) and oxidative stress and increasing NO bioavailability in an adenosine monophosphate-activated protein kinase/peroxisome proliferator-activated receptor δ (AMPK/PPARδ) pathway-dependent manner (Cheang et al., 2014).

Brain-derived neurotrophic factor (BDNF), originally discovered in the brain as a type of neurotrophin, is known to have crucial neurotrophic functions in the brain and peripheral nerves that affect neural development, survival, and repair after injury (Genzer et al., 2016). Interestingly, treatment with metformin increases BDNF levels in mice with Parkinson’s disease (Patil et al., 2014). Vascular endothelial cells synthesize and secrete BDNF (Nakahashi et al., 2000), which prolongs endothelial cell survival through tropomyosin-related kinase receptor (TrK) (Caporali and Emanueli, 2009). Decreased circulating BDNF levels were observed in patients with T2DM (Krabbe et al., 2007). Cerebrovascular BDNF protein was reduced in the cortical endothelium in diabetic rats (Navaratna et al., 2011). Endothelial progenitor cell (EPC) transplantation and RWJ administration *in vivo* promotes angiogenesis and neurogenesis after diabetic stroke with increased expression of vascular endothelial growth factor (VEGF) and BDNF (Bai et al., 2015). BDNF ameliorates endothelial cell dysfunction by promoting neovascularization, modulating endothelial nitric oxide production, and inhibiting apoptosis (Nakamura et al., 2006; Meuchel et al., 2011; Takeda et al., 2013).

Revealing the molecular mechanism by which metformin exerts its beneficial effect in endothelial cells is an intriguing endeavor. Considering the protective role of BDNF in the cardiovascular system, we explored the protective relationship between metformin and BDNF. By using a HG-induced endothelial injury model, our study aims to provide a novel mechanism by which the established antihyperglycemic drug, metformin, offers, endothelial protection.

## Materials and Methods

### Cell Culture and Ethics Statement

This study was approved by the Medical Ethics Committee for Clinical Research of Zhongda Hospital Affiliated to Southeast University. Written informed consent was obtained from all subjects prior to the study.

Human umbilical vein endothelial cells (HUVECs) were obtained from five human donors. Umbilical cords (10–15 cm) were cut soon after birth and stored in phosphate buffered saline (PBS) immediately. The endothelial cells were obtained from filling the lumens of human umbilical veins with collagen enzyme solution, and then, the cells were cultured in endothelial cell medium (ECM) (ScienCell, United States). The cells were incubated at 37°C in 5% CO_2_ and maintained using standard cell culture techniques. After reaching a confluence of 80%, the cells were detached using 0.25% trypsin–EDTA. The cells in this experiment were used within three to four passages.

The HG-induced injury model was established using a method described in previous studies. Cells were incubated in ECM with 5.5 mmol/l (euglycemia) or 33.3 mmol/l (hyperglycemia) glucose (Sigma-Aldrich, United Kingdom) for 24 h (Zhu et al., 2017). Different concentrations of mannitol were used for osmotic pressure control, and no effects were observed. When cells were confluent, they were serum-starved. Then HUVECs were pretreated in basal glucose medium with or without 0.01 mmol/l metformin (HG + MET treatment) (Merck, Germany) for 1 h (Bakhashab et al., 2016).

To explore the roles played by AMPK, CREB, and BDNF in the protective effects of metformin, HUVECs were also pretreated with or without 10 μM compound C (CC) (Selleckchem, United States), 25 μM 2-naphthol-AS-E-phosphate (KG-501, Sigma Chemical Co., Ltd., PR China), or 1 μg/ml TrkB-Fc chimera (R&D Systems, United States) at the same time as adding metformin, and cells were then incubated for 24 h in the presence of HG concentration.

CC has been widely used as a type of pharmacological AMPK inhibitor (McCullough et al., 2005). KG-501 is a small molecule inhibitor of cyclic AMP response element (CRE)-binding protein (CREB) (Sun et al., 2008), and TrkB-Fc chimeric protein serves as a BDNF scavenger (Huang et al., 2010). The inhibitor concentrations were referenced in previous studies (Helan et al., 2014; Kang et al., 2014; Zhou et al., 2017). According to the manufacturer’s instructions, metformin, D-glucose, and TrkB-Fc were dissolved in sterile PBS, and CC and KG-501 were dissolved in dimethylsulfoxide (DMSO).

### Cell Viability and Proliferation Assays

For the Cell Counting Kit-8 (CCK8) cell viability assay, HUVECs were seeded into 96-well cell culture plates (5000 cells/well). Following the manufacturer’s instructions for the CCK8 cell viability assay (Beyotime Institute of Biotechnology, China), the cells were incubated with the CCK8 reagent at 37°C for 2 h, and the absorbance at 450 nm was measured using a microplate reader (STNERGY/H4, BioTek, United States).

For the 5-bromo-2′-deoxyuridine (BrdU) incorporation assay, a BrdU incorporation assay kit (Roche, United States) was used to assess HUVEC proliferation. The HUVECs were seeded in 96-well cell culture plates at a density of 2 × 10^3^ cells per well. After 12 h, the cells were incubated in FBS-free ECM for 6 h and then in ECM with 5.5 or 33.3 mmol/l glucose for 24 h. The cells in the metformin and metformin plus inhibitor treatment groups were incubated with metformin before the HG treatment. After 24 h, the BrdU–labeling solution (10 μM) was added to the cells, followed by a 12 h incubation. After the DNA was denatured, a peroxidase-labeled anti-BrdU monoclonal antibody was added to the cells, and the samples were incubated at room temperature for 90 min. The absorption of the BrdU-antibody complexes at dual wavelengths of 450/595 nm was measured. All samples used in the experiments contained 0.03% DMSO, and the presence of DMSO did not affect HUVEC proliferation.

### Apoptosis Analysis

Flow Cytometry Analysis Cell apoptosis was assessed in HUVECs using the Annexin V-FITC/propidium iodide (PI) apoptosis detection kit (Miltenyi Biotec, Germany). Cells from each group were collected, rinsed with cold PBS, and stained using Annexin V-FITC/PI. The cells were immediately detected using an Accuri C6 flow cytometry (BD Biosciences, United States). Data were analyzed using FlowJo software (ver. 10.0, FlowJo LLC, United States).

TUNEL assays cell apoptosis was also evaluated using a transferase-mediated dUTP nick-end labeling (TUNEL) method with a detection kit (Roche, Germany) following the manufacturer’s instructions. The cells were fixed with 4% paraformaldehyde, rinsed with PBS, and then permeabilized with 0.1% Triton X-100 for FITC end-labeling the fragmented DNA of the apoptotic HUVECs. The FITC-labeled TUNEL-positive cells were imaged under a fluorescent microscopy (IX-71; Olympus, Japan).

### Enzyme-Linked Immunosorbent Assay

Enzyme-Linked Immunosorbents (ELISAs) (Promega, United States) were performed according to the manufacturer’s protocols to measure the BDNF levels. The supernatant of each sample was collected and stored at 70°C for further use. The diluted standards or samples were added into each well, and the plate was incubated for 1 h at 37°C. The reacted plate was rinsed, incubated for 30 min at 37°C with the indicated antibodies and then rewashed. The reactions were terminated with stop solution, and the optical density (OD) at 450 nm was measured with a microplate reader. The results were standardized to the cell number in each group.

### Western Blotting

Cells were lysed in lysis buffer (Beyotime Institute of Biotechnology, China) supplemented with 1 mM PMSF with or without 0.01 mM phosphatase inhibitors. The protein concentration was determined using the BCA protein assay (Beyotime Institute of Biotechnology, China). Twenty micrograms of protein from each sample was separated by 12% or 15% SDS-PAGE and electrotransferred onto PVDF membranes (Millipore, United States) for immunoblotting analysis. The following primary antibodies were used: anti-BDNF (1:500, ab108319, Abcam), anti-CREB (1:500, 12208-1-AP, Proteintech), anti-pCREB (Ser133) (1:500, AF3189, Affinity), and anti-beta-actin (1:1000, ab6276, Abcam). Beta-actin was used as the internal reference. Antibodies against total or phosphorylated AMPK [anti-AMPKα, anti-pAMPKα (Thr172)] were obtained from Cell Signaling Technology (Beverly, United States). After incubating with the appropriate HRP-conjugated secondary antibody, proteins were detected using a ChemiDoc XRS imaging system and were analyzed in ImageJ software.

### Quantitative Polymerase Chain Reaction (q-PCR) Assay

Total RNA from the cells was extracted using TRIzol reagent (Invitrogen, United States) according to the manufacturer’s protocol. A UV spectrophotometer was used to measure the purity and concentration of the RNA, and agarose gel electrophoresis was used to verify the RNA integrity. The cDNA templates were synthesized using the PrimeScript^TM^ RT reagent kit with gDNA Eraser (TaKaRa, Japan) according to the manufacturer’s instructions. The qPCR analysis was performed using IQ SYBR Green Supermix (Bio-Rad, United States) and the Bio-Rad MJ Mini Opticon Real-Time PCR System, along with Bio-Rad CFX Manager analysis software. The resulting amplification and melting curves were analyzed to identify the specific PCR products. The relative gene expression values were calculated using the comparative 2^-ΔΔCt^ method, and GAPDH was used as control. The PCR primers for BDNF were as follows: forward primer: 5′-GGGACCCGTGAGTTTGTGT-3′, and reverse primer: 5′-TTGCTTCTTTCATGGGGGCA-3′. The PCR primers for GAPDH were as follows: forward primer: 5′-GACAGTCAGCCGCATCTTCT-3′, and reverse primer: 5′-GCGCCCAATACGACCAAATC-3′.

### Chromatin Immunoprecipitation Assay

Chromatin immunoprecipitation assay (ChIP) assays were performed as prescribed previously with minor modifications (Madhu et al., 2018). Cells after treatment were fixed with 1% formaldehyde and were washed twice with 1^∗^ PBS. The cross-linking reaction was terminated by incubation in 0.125 M glycine at room temperature for 5 min. Then, a ChIP Assay kit (Millipore) was used to perform the ChIP assay. In brief, the cells were harvested and sonicated. Equal volumes (10 μl) of cell products were taken out as a control. The others were incubated with CREB antibodies at 4°C overnight. The next day, the mixture was incubated with Protein A/G beads at room temperature for 2 h. Then, the beads were washed, and the DNA was isolated. qRT-PCR was performed to measure the DNA abundance in the different groups. IgG and CREB antibodies were purchased from Proteintech. DNA samples were further used to perform PCR analyses to confirm the binding of CREB on the BDNF promoter. The primer sequences used for ChIP PCR were forward primer: 5′-GCGCTGAATTTTGATTCTGGTAAT-3′, and reverse primer: 5′-AATGGGAAAGTGGGTGGGAG-3′.

### Plasmid Construction

The overexpressed BDNF constructs were based on the pEGFP-N1 vector (Clontech, United States). The full-length human BDNF complementary DNA (cDNA) sequence (GenBank accession No. X91251.1) was obtained via PCR and then inserted into the NheI and BamHI sites of the pEGFP-N1 vector (Clontech, United States). The PCR were performed using the following conditions: 95°C for 5 min; 32 cycles of 95°C for 30 s, 60°C for 30 s, and 72°C for 2 mins; 72°C for 10 mins. The primers used were as follows: the forward primer F: 5′-CGGGCTAGCCATGACCATCCTTTTCCTTACTA-3′, and reverse primer R: 5′-CGGGATCCTCTTCCCCTTTTAATG GTCAAT-3′. The primers were designed by Sangon Biotech (Shanghai, China).

### Statistical Analysis

Data were analyzed using the statistical software SPSS (v.13.0.0; SPSS Inc., United States) and are presented as the means ± standard deviation. All experiments were repeated a minimum of three times. Two-tailed Student’s *t*-tests and one-way ANOVA were used to compare the results. The results were considered statistically significant at *p* < 0.05.

## Results

### High Glucose Reduced Cell Proliferation and Induced Cell Apoptosis via Changes in BDNF Expression

**FIGURE 1:**
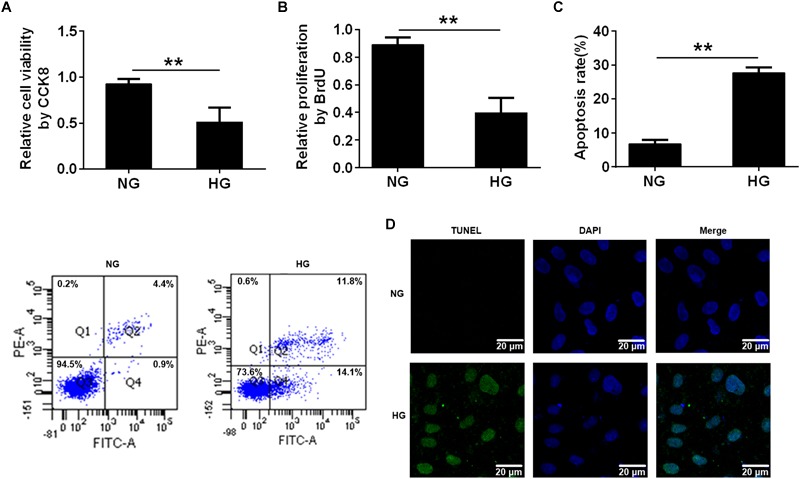
High glucose reduced cell proliferation and induced cell apoptosis. **(A)** CCK8 assay for cell viability in HUVECs treated with NG (5.5 mmol/l) and HG (33.3 mmol/L) for 24 h. **(B)** BrdU incorporation assay of cell proliferation in HUVECs treated with NG and HG. **(C)** Annexin V/PI assay of cell apoptosis in HUVECs treated with the NG and HG conditions. The histograms represent the apoptosis rates in the two groups. **(D)** Representative photographs of TUNEL staining in the different groups. The results are expressed as the means ± SD. ^∗∗^*p* < 0.01; two-tailed Student’s *t*-test.

Previous studies have demonstrated that HG caused endothelial cell dysfunction (Sheu et al., 2005), so we applied a CCK8 assay and a BrdU incorporation assay to determine the effects of HG on cell viability and proliferation. The CCK8 assay revealed that cell viability was reduced in the HG-treated HUVECs compared with the normal glucose (NG)-treated HUVECs (^∗∗^*p* < 0.01, Figure 1A). Similarly, the BrdU assay showed that HG reduced cell proliferation compared with the control group (NG) (^∗∗^*p* < 0.01, Figure 1B). Furthermore, flow cytometry results revealed that incubating HUVECs with HG induced cell apoptosis (^∗∗^*p* < 0.01, Figure 1C). It was also determined that there were more TUNEL^+^ HUVECs in the HG-treated group than in the control group (Figure 1D). These results demonstrated that HG reduced cell proliferation and induced cell apoptosis.

**FIGURE 2:**
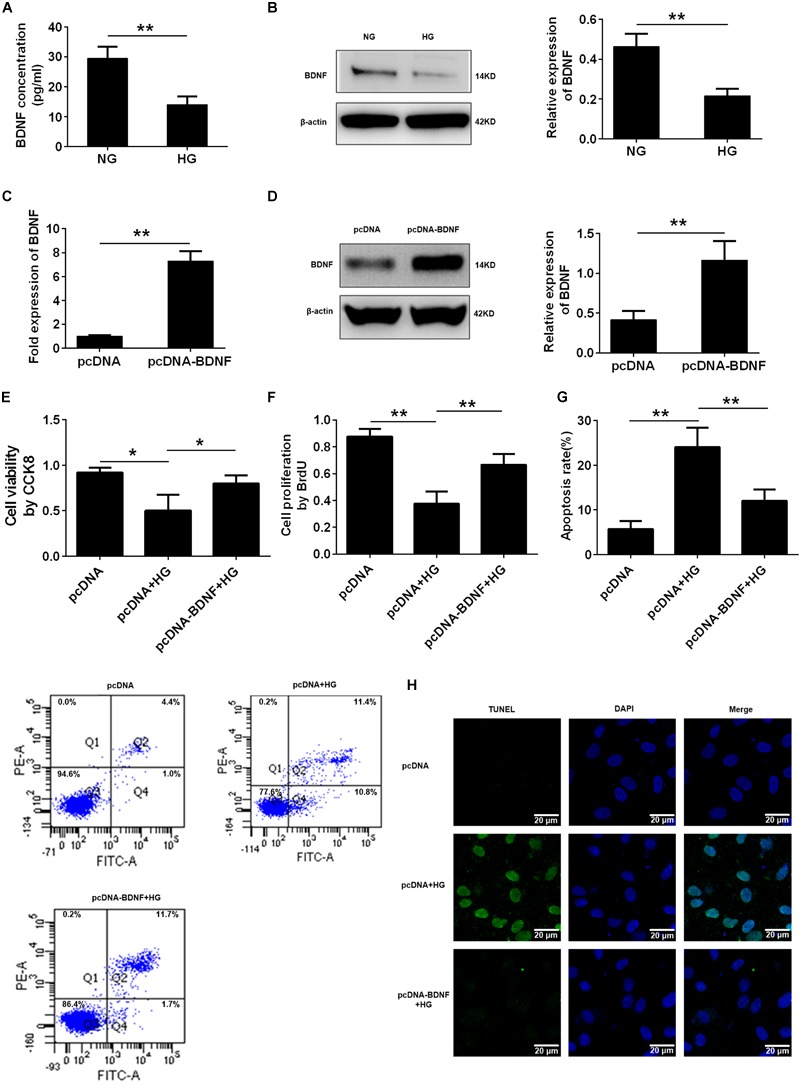
BDNF was involved in high glucose-induced cell proliferation and apoptosis. **(A)** ELISA analysis of BDNF expression in HUVECs treated with NG and HG (33.3 mmol/l). **(B)** Western blot analysis of BDNF expression in HUVECs treated with the NG and HG conditions. The histograms represent the relative BDNF intensity. **(C)** q-PCR analysis of BDNF expression in HUVECs overexpressing BDNF. **(D)** Western blot analysis of BDNF expression in HUVECs overexpressing BDNF. The histograms represent the relative BDNF intensity. **(E)** CCK8 assay in HUVECs treated with pcDNA, pcDNA + HG, and pcDNA-BDNF + HG for 24 h. **(F)** BrdU incorporation assay in HUVECs treated with pcDNA, pcDNA + HG, and pcDNA-BDNF + HG. **(G)** Annexin V/PI assay in HUVECs treated with pcDNA, pcDNA + HG, and pcDNA-BDNF + HG. The histograms represent the apoptosis rates in the different groups. **(H)** Representative photographs of TUNEL staining in the different groups. The results are expressed as the means ± SD. ^∗^*p* < 0.05, ^∗∗^*p* < 0.01; two-tailed Student’s *t*-test and one-way ANOVA.

Recently, our clinical research from patients with stable coronary artery disease showed that the plasma BDNF levels were strongly associated with the presence of diabetes mellitus (Jin et al., 2018), so we explored the BDNF expression of HG- injured HUVECs. We treated the HUVECs with different concentrations of glucose (5.5, 15, 33.3, and 60 mmol/L), and mannitol (0, 9.5, 27.8, and 54.5 mmol/L) as osmotic pressure control. And we used ELISA and Western blotting to detect BDNF expression in endothelial cells. The ELISA results revealed that BDNF was secreted into the cell-conditioned medium, and both ELISA and Western blotting assays illustrated that exposure of HUVECs to HG resulted in a significant decrease in BDNF protein expression, especially in 33.3 mmol/L and 60 mmol/L glucose conditions, compared with HUVECs that had been cultured for the same period of time in NG. However, no alteration was observed in the cells treated with different concentrations of mannitol (^∗∗^*p* < 0.01, Figures 2A,B and Supplementary Figure S2). To further ascertain whether the BDNF level might affect the HG-induced cell proliferation and apoptosis, we determined the effects of BDNF overexpression (^∗∗^*p* < 0.01, Figures 2C,D) on cell proliferation and apoptosis in HG-injured HUVECs. CCK8 and BrdU assays found that overexpressed BDNF could alleviate the HG-induced repression of cell viability and proliferation (^∗^*p* < 0.05, ^∗∗^*p* < 0.01, Figures 2E,F). Furthermore, flow cytometry and TUNEL staining data showed that BDNF overexpression markedly reduced HG-induced cell apoptosis (^∗∗^*p* < 0.01, Figures 2G,H). Taken together, we found that HG reduced cell proliferation and induced cell apoptosis by affecting BDNF expression.

### The Effects of Metformin on HUVEC Cell Proliferation and Apoptosis in Response to HG Treatment

**FIGURE 3:**
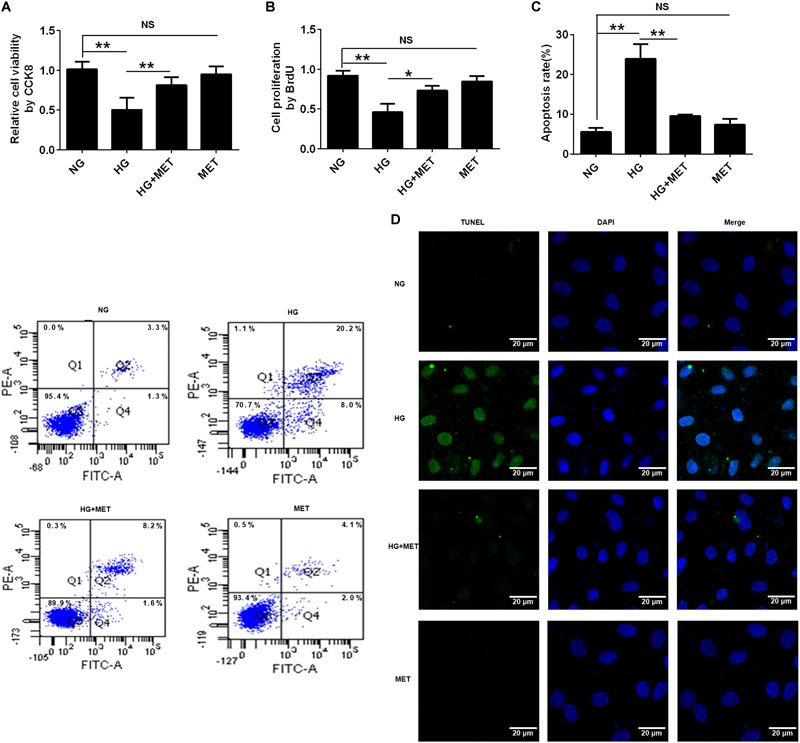
The effects of metformin on HUVEC cell proliferation and apoptosis in response to HG treatment. **(A)** CCK8 assay in HUVECs treated with NG, HG (33.3 mmol/l), HG plus 0.01 mmol/l metformin (HG + MET), and MET (0.01 mmol/l) conditions. **(B)** BrdU incorporation assay in HUVECs treated with NG, HG, HG + MET, and MET. **(C)** Annexin V/PI assay in HUVECs treated with NG, HG, HG + MET, and MET. The histograms represent the apoptosis rates in the four groups. **(D)** Representative photographs of TUNEL staining in the different groups. The results are expressed as the means ± SD. ^∗^*p* < 0.05, ^∗∗^*p* < 0.01; NS, not significant; one-way ANOVA.

Previous studies have reported that metformin improves HG-induced vascular injury. To determine the effect of metformin on the viability and proliferation of HG-incubated HUVECs, we used a CCK8 assay and conducted a BrdU incorporation assay. The results of the CCK8 assay showed that HG reduced cell viability compared with the control group (NG) (^∗∗^*p* < 0.01, Figure 3A). However, the metformin treatment rescued the viability of HG-treated cells, while the metformin group had no difference from NG (^∗∗^*p* < 0.01, NS *p* > 0.05, Figure 3A). Similarly, HUVECs proliferation was reduced in the HG condition compared with the NG condition, and metformin rescued the HG-induced reduction in proliferation, identified via the BrdU assay (^∗^*p* < 0.05, ^∗∗^*p* < 0.01, NS *p* > 0.05, Figure 3B). Furthermore, the cell apoptosis flow cytometry and TUNEL staining results revealed that incubating HUVECs with HG induced cell apoptosis, which was significantly suppressed by the metformin treatment (^∗∗^*p* < 0.01, NS *p* > 0.05, Figures 3C,D). Taken together, these results demonstrated that metformin improved cell proliferation and alleviated HUVEC apoptosis under HG conditions.

### Metformin Improved Cell Proliferation and Ameliorated Cell Apoptosis by Modulating BDNF Expression

**FIGURE 4:**
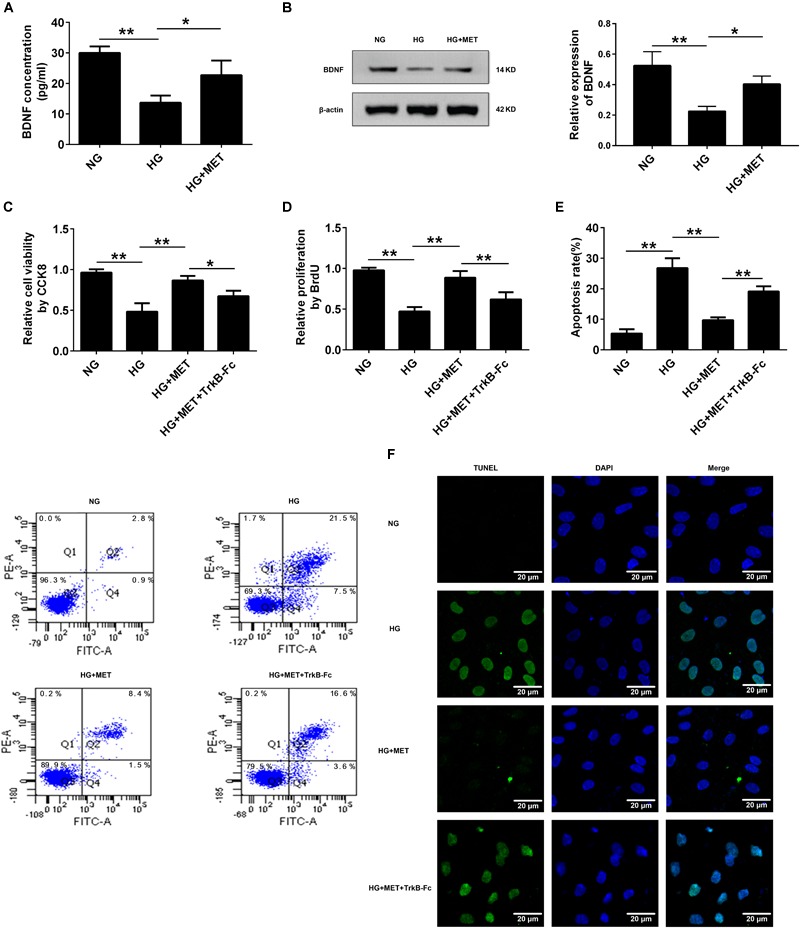
Metformin improved cell proliferation and ameliorated cell apoptosis by modulating BDNF expression. **(A)** ELISA analysis of BDNF expression in HUVECs treated with NG, HG (33.3 mmol/l) and HG + MET (0.01 mmol/l) conditions. **(B)** Western blot analysis of BDNF expression in HUVECs treated with NG, HG, and HG + MET. The histograms represent the relative BDNF intensity. **(C)** CCK8 assay in HUVECs treated with the NG, HG, HG + MET, and HG + MET plus 1 μg/ml TrkB-Fc (HG + MET + TrkB-Fc) for 24 h. **(D)** BrdU incorporation assay in HUVECs treated with NG, HG, HG + MET, and HG + MET + TrkB-Fc. **(E)** Annexin V/PI assay of cell apoptosis in HUVECs treated with the NG, HG, HG + MET, and HG + MET + TrkB-Fc conditions. The histograms represent the apoptosis rates in the four groups. **(F)** Representative photographs of TUNEL staining in the different groups. The results are expressed as the means ± SD. ^∗^*p* < 0.05, ^∗∗^*p* < 0.01; one-way ANOVA.

We proposed that BDNF might be involved in the protective effects of metformin on HG-injured HUVECs. To test our hypothesis, we used ELISA and Western blotting to detect BDNF expression in HG-incubated cells treated with or without metformin. The ELISA results revealed that compared with the HG treatment, metformin treatment upregulated BDNF protein expression (^∗^*p* < 0.05, Figure 4A). In addition to the ELISA results, the Western blotting results revealed that BDNF expression was preserved in the HG-incubated HUVECs subjected to the metformin treatment (^∗^*p* < 0.05, Figure 4B). These results indicated that metformin induced BDNF expression, and BDNF may play a role in metformin-mediated endothelial protection under HG conditions.

To elucidate the potential function of BDNF in HUVECs incubated with HG and treated with metformin, we used a soluble BDNF neutralizer, TrkB-Fc, to reduce the effects of BDNF. Interestingly, we found that the protective effects of metformin on HUVEC proliferation and apoptosis were blunted by neutralizing the secreted BDNF with the TrkB-Fc chimera (^∗^*p* < 0.05, ^∗∗^*p* < 0.01, Figures 4C–F). Taken together, these results demonstrated that metformin improved cell proliferation and ameliorated cell apoptosis by modulating BDNF expression.

### BDNF Expression Was Regulated by Metformin Through CREB Activation

To further define the metformin effects, the transcriptional pathway involved in metformin-induced BDNF expression, the CREB pathway, which has been implicated in BDNF induction, was identified as a potential candidate (Tao et al., 1998).

**FIGURE 5:**
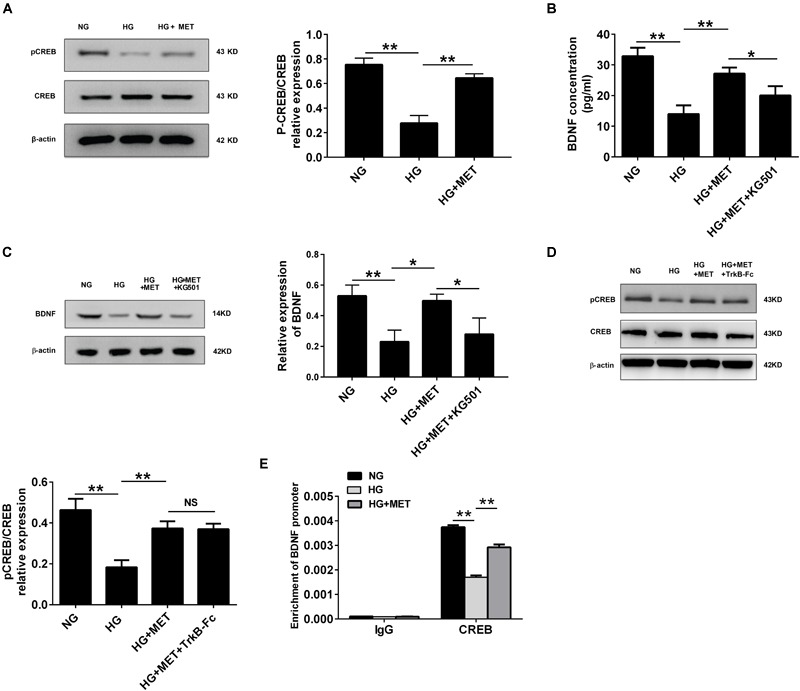
BDNF expression was regulated by metformin through CREB activation. **(A)** Western blot analysis of CREB and pCREB expression in HUVECs treated with NG, HG (33.3 mmol/l), and HG + MET (0.01 mmol/l) conditions. Histograms represent the relative pCREB/CREB intensity. **(B)** ELISA analysis of BDNF expression in HUVECs treated with the NG, HG, HG + MET, and HG + MET plus 25 μM KG501 (HG + MET + KG501) conditions for 24 h. **(C)** Western blot analysis of BDNF expression in HUVECs treated with NG, HG, HG + MET, and HG + MET + KG501. Histograms represent the relative BDNF intensity. **(D)** Western blot analysis of CREB and pCREB expression in HUVECs treated with NG, HG, HG + MET, and HG + MET + TrkB-Fc. Histograms represent the relative pCREB/CREB intensity. **(E)** Detection of CREB binding in BDNF promoter regions by chromatin immunoprecipitation (ChIP) assay in HUVECs. Human IgG was used as a negative control. The results are expressed as the means ± SD. ^∗^*p* < 0.05, ^∗∗^*p* < 0.01; NS, not significant; one-way ANOVA.

We performed Western blotting to detect pCREB and CREB expression in HG-incubated cells treated with or without metformin. The results revealed that pCREB expression was significantly reduced in the HG-incubated HUVECs (^∗∗^*p* < 0.01, Figure 5A), which was reversed by the metformin treatment (^∗∗^*p* < 0.01, Figure 5A). However, we did not observe any significant changes in CREB expression among the three groups (Figure 5A).

Furthermore, we used a CREB inhibitor, KG-501, to determine whether the regulatory effect of metformin-mediated CREB activation influences BDNF expression. ELISA and Western blotting assays indicated that BDNF expression was lower in the HG-treated group than in the NG group, while the HG plus MET treatment alleviated the reduced BDNF expression. Additionally, compared with the HG + MET-incubated HUVECs, the HUVECs treated with KG-501 presented with a reduced BDNF level (^∗^*p* < 0.05, ^∗∗^*p* < 0.01, Figures 5B,C).

Also, we tested the effect of BDNF inhibitor on CREB activation, and the results of Western blotting indicated that HG + MET-incubated HUVECs with or without TrkB-Fc had no significant difference in CREB and pCREB expression (Figure 5D). These results demonstrated that metformin modulated BDNF expression through CREB phosphorylation in HG-incubated HUVECs.

In addition to determine whether CREB transcription factor physically interacted with the CRE sites within the BDNF promoter, a ChIP assay was performed. Immunoprecipitation of cross-linked chromatin from HUVECs with anti-CREB antibody followed by PCR amplification of the region illustrated that CREB protein selectively binds to this region of the BDNF promoter in exon III. Taken together, our study found that BDNF expression was regulated by metformin under HG conditions through CREB, which could bind directly to the promoter regions of BDNF (^∗∗^*p* < 0.01, Figure 5E).

### AMPK Was Involved in CREB/BDNF Regulation in HG-Incubated HUVECs Treated With Metformin

**FIGURE 6:**
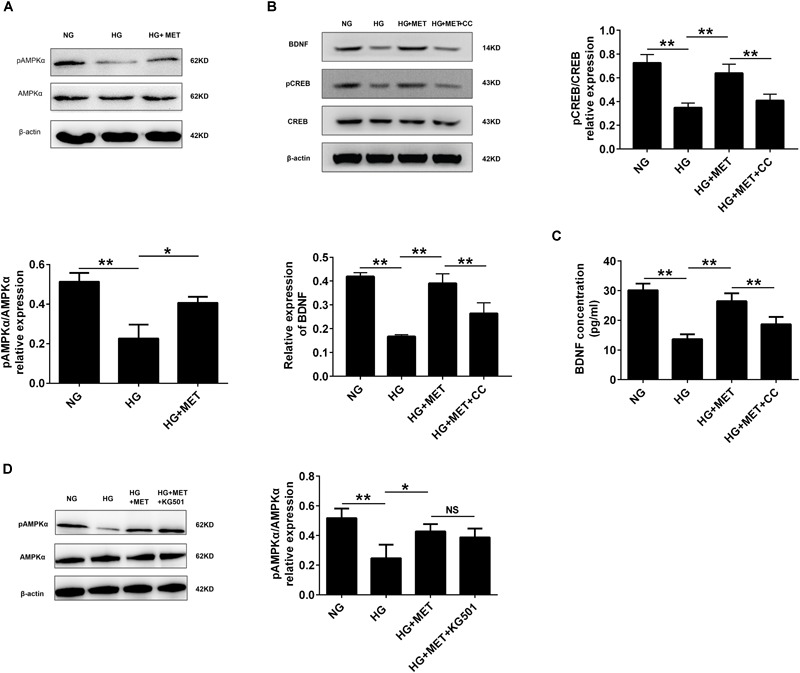
AMPK was involved in CREB/BDNF regulation in high glucose-incubated HUVECs treated with metformin. **(A)** Western blot analysis of AMPKα and pAMPKα expression in HUVECs treated with NG, HG (33.3 mmol/l), and HG + MET (0.01 mmol/l) conditions. Histograms represent the relative intensity of pAMPKα/AMPKα. **(B)** Western blot analysis of CREB, pCREB, and BDNF expression in HUVECs treated with the NG, HG, HG + MET, and HG + MET plus 10 μM CC (HG + MET + CC) conditions. Histograms represent the relative intensity of pCREB/CREB and BDNF. **(C)** ELISA analysis of BDNF expression in HUVECs treated with NG, HG, HG + MET, and HG + MET + CC. **(D)** Western blot analysis of AMPKα and pAMPKα expression in HUVECs treated with NG, HG, HG + MET, and HG + MET + KG501. Histograms represent the relative intensity of pAMPKα/AMPKα. The results are expressed as the means ± SD. ^∗^*p* < 0.05, ^∗∗^*p* < 0.01; NS, not significant; one-way ANOVA.

Previous studies have documented that AMPK modulates pulmonary artery endothelial cell angiogenesis (Teng et al., 2013), and that metformin activates AMPK (Hattori et al., 2006). Therefore, we wondered whether AMPK was involved in CREB/BDNF regulation in HG + MET-incubated HUVECs. We used Western blotting to detect the AMPKα and pAMPKα protein levels. The results revealed that the pAMPKα protein level was significantly decreased in the HG group, while metformin increased pAMPKα expression (^∗^*p* < 0.05, ^∗∗^*p* < 0.01, Figure 6A). These results demonstrated that metformin induced AMPK activation.

Since metformin affects CREB/BDNF expression and influences AMPK activation, AMPK function on CREB phosphorylation and BDNF expression was evaluated in the HG + MET-treated HUVECs. The Western blotting results revealed that CC, a common AMPK inhibitor, reversed the metformin-induced upregulation of pAMPK, pCREB and BDNF expression in HG-incubated HUVECs, but no significant change was observed in AMPK and CREB expression (^∗∗^*p* < 0.01, Figure 6B and Supplementary Figure S3). As expected, the ELISA results also demonstrated that CC repressed the metformin-induced BDNF upregulation in HG-incubated HUVECs (^∗∗^*p* < 0.01, Figure 6C). We also employed A-769662, AICAR and siAMPK to explore the role of AMPK more specifically and the results further showed that AMPK specific activation increased the expression of pCREB and BDNF, while siAMPK inhibited pCREB and BDNF expression (^∗∗^*p* < 0.01, Supplementary Figure S1).

Furthermore, we explored the effect of CREB inhibitor on AMPK activation. However, the Western blotting results indicated that HG + MET-incubated HUVECs with or without KG-501 had no significant difference in AMPK and pAMPK expression (Figure 6D).

These results demonstrated that AMPK played a role in regulating CREB/BDNF expression in HG + MET-treated HUVECs.

### AMPK Was Involved in the Metformin-Induced Cell Proliferation and Apoptosis of HG-Incubated HUVECs

**FIGURE 7:**
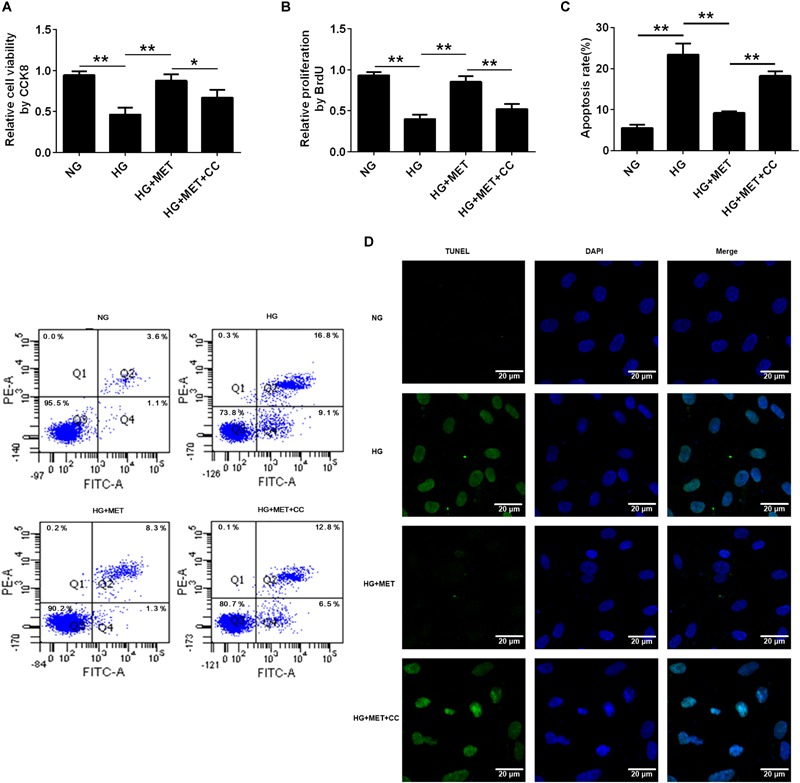
AMPK was involved in the metformin-induced cell proliferation and apoptosis of HG-incubated HUVECs. **(A)** CCK8 assay in HUVECs treated with NG, HG (33.3 mmol/l), HG + MET (0.01 mmol/l), and HG + MET + CC (10 μM) conditions. **(B)** BrdU incorporation assay in HUVECs treated with NG, HG, HG + MET, and HG + MET + CC. **(C)** Annexin V-PI assay in HUVECs treated with NG, HG, HG + MET, and HG + MET + CC. The histograms represent the apoptosis rates in the four groups. **(D)** Representative photographs of TUNEL staining in the different groups. The results are expressed as the means ± SD. ^∗^*p* < 0.05, ^∗∗^*p* < 0.01; one-way ANOVA.

Since both metformin and AMPK regulate cell proliferation and apoptosis, CCK8, BrdU, flow cytometry, and TUNEL staining assays were used to evaluate cell proliferation and apoptosis to assess the effects of an AMPK inhibitor on HG-incubated HUVECs. CCK8 and BrdU assays indicated that the AMPK inhibitor reversed the cell viability and proliferation of HG + MET-treated HUVECs (^∗^*p* < 0.05, ^∗∗^*p* < 0.01, Figures 7A,B). The flow cytometry cell apoptosis and TUNEL staining results revealed that the HG conditions increased HUVECs apoptosis and that this effect was reversed by metformin treatment. However, the AMPK inhibitor impaired the effects of metformin on HG-incubated HUVECs (^∗∗^*p* < 0.01, Figures 7C,D).

**FIGURE 8:**
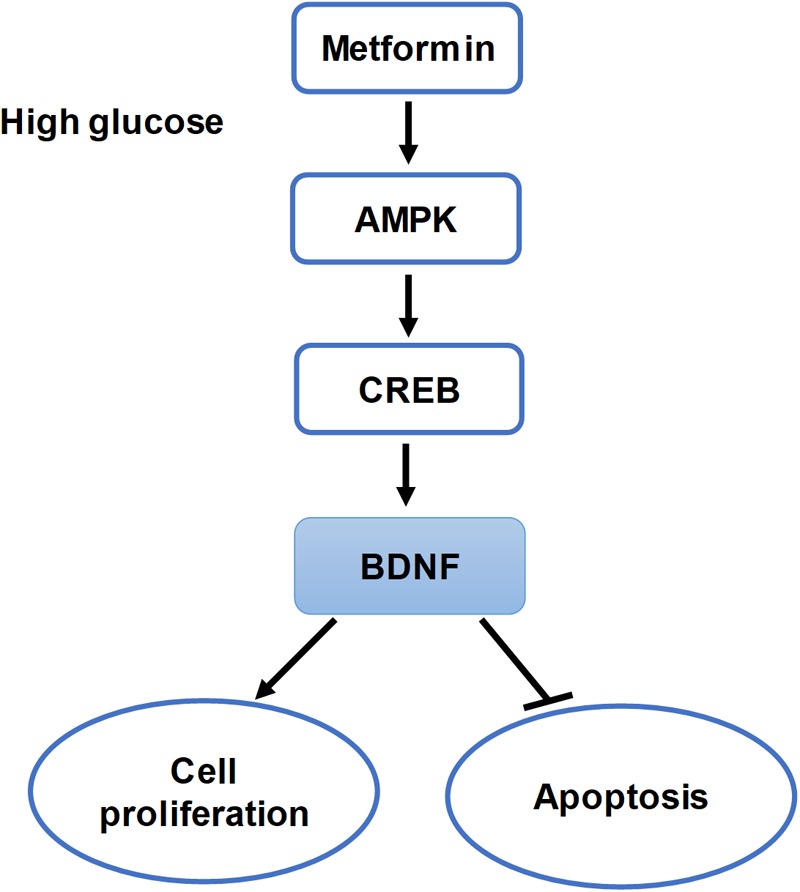
Summary of the hypothesis of the study. Schematic of this study that metformin improves cell proliferation and ameliorates cell apoptosis via the AMPK/CREB/BDNF pathway in HG-incubated HUVECs.

The above results all indicated that AMPK activation by metformin affected cell proliferation and apoptosis via regulating CREB/BDNF expression in HG-incubated HUVECs (Figure 8).

## Discussion

This study revealed that metformin significantly improved endothelial cell proliferation and inhibited apoptosis under HG conditions through a novel mechanism involving the AMPK/CREB/BDNF pathway. Our research showed that HG injury reduced HUVEC proliferation and increased apoptosis. Interestingly, BDNF expression was also reduced in HG-incubated HUVECs and that BDNF overexpression could reverse the reduced proliferation and increased apoptosis in HG-injured HUVECs. However, metformin treatment increased BDNF expression and reversed the HG injury effects. Furthermore, metformin-induced BDNF expression was AMPK-dependent and required the activation of the downstream target CREB.

As described previously, HG leads to endothelial cell dysfunction and metformin improves the endothelial function of patients with T2DM and reduces mortality and cardiovascular end points of patients with T2DM and CVD (1998; Mather et al., 2001; Sheu et al., 2005; Roussel et al., 2010; Aguilar et al., 2011). In the present study, we also identified that HG conditions reduced HUVEC proliferation and increased cell apoptosis, while metformin improved cell survival and inhibited apoptosis (Figures 1–3). Multiple studies have discussed the mechanisms underlying the cardiovascular protective effects of metformin. Metformin protects endothelial cells from HG-induced premature senescence by restoring the HG-induced reduction in SIRT1 expression (Arunachalam et al., 2014) and attenuates atherosclerosis development by reducing the levels and translocation of dynamin-related protein (Drp1), preventing mitochondrial fragmentation (Wang et al., 2017). Furthermore, our study explored a novel mechanism by which metformin improves HG-induced endothelial dysfunction through the BDNF pathway.

Although BDNF is a well-characterized trophic factor for neurons, it is also essential for cardiovascular development and for CVD and CVD-related disorders. The embryonic hearts from BDNF^-/-^animals exhibit both atrial septal defects and intramyocardial hemorrhages. Conversely, BDNF overexpression in midgestational mouse hearts increases capillary density, establishing an essential role for BDNF in modulating cardiac microvascular ECs during development (Donovan et al., 2000). In addition, BDNF not only acts on endothelial cells to promote neovascularization under hypoxia but also stimulates human microvascular endothelial cell migration via a process involving the TrkB/ERK/integrin α(V)β(3)/FAK pathway(Nakamura et al., 2006; Matsuda et al., 2012). TrkB activation by BDNF protects endothelial integrity during atherogenesis by promoting Ets1-mediated VE-cadherin expression (Jiang et al., 2015). These studies indicate that BDNF plays an important role in endothelial cell function. Our study found that BDNF expression was reduced in HG-injured HUVECs with reduced cell proliferation and increased apoptosis levels, while BDNF overexpression reversed these effects (Figure 2 and Supplementary Figure S2). However, metformin increased BDNF expression, improved cell proliferation, and alleviated HUVEC apoptosis under HG conditions, suggesting that metformin induced BDNF expression to protect endothelial cells under HG conditions. We further found that the protective effects of metformin were inhibited by TrkB-Fc treatment, a soluble BDNF neutralizer (Figures 3, 4). Arunachalam et al. (2014) demonstrated that metformin protects endothelial cells from HG-induced premature senescence by attenuating a reduction in SIRT1 expression and the levels of downstream targets, including FoxO-1 and p53/p21. In the present study, metformin improved cell proliferation and ameliorated cell apoptosis by regulating BDNF expression. These different downstream signaling pathways may function in parallel or interact with each other, which will be investigated in our future research.

The transcription factor CREB is implicated in glucose homeostasis, growth-factor-dependent cell survival, learning, and memory. Phosphorylation regulates CREB activation (Mayr and Montminy, 2001). CREB mediates cAMP-responsive gene activation by binding as a dimer to a conserved cAMP-responsive element (CRE), TGACGTCA. CREB phosphorylation at Ser133 recruits the transcriptional co-activator CREB-binding protein (CBP) and its paralogue p300 (Shaywitz and Greenberg, 1999). Several studies have noted a potential role for CREB in maintaining cardiac and vascular function. In mice, the cardiac-specific expression of dominant-negative CREB increases oxidative stress and mortality (Watson et al., 2010), while loss of aortic CREB expression has been found in rodent models of hypertension, atherosclerosis, and insulin resistance (Schauer et al., 2010). In rodent models of insulin-resistant and insulin-deficient diabetes, the levels of CREB and the active form of CREB (pCREB) are decreased in medial vascular smooth muscle cells (VSMCs) (Watson et al., 2001). However, only a few studies are available on the interaction between CREB and BDNF in the cardiovascular system. Our present data revealed that both pCREB and BDNF expression levels were decreased in HG-incubated HUVECs, while metformin reversed pCREB and BDNF downregulation in HG-incubated HUVECs, demonstrating a consistent regulatory effect of metformin on pCREB and BDNF levels. Furthermore, a previous study revealed that pCREB might regulate BDNF transcription (Williams et al., 2008). Thus, in this study, we found that CREB bound directly to the promoter regions of BDNF and that CREB inhibition blocked metformin-induced BDNF upregulation in HG-incubated HUVECs, while BDNF inhibition had no significant influence on pCREB expression (Figure 5). In summary, our study provided additional evidence that BDNF expression is modulated by metformin through CREB activation.

AMPK is a phylogenetically conserved heterotrimer protein consisting of three subunits, α, β, and γ, each of which has at least two isoforms (Davis et al., 2006). Two different isoforms (α1 and α2) of the α subunit are differentially expressed in different tissues, and endothelial cells express both subunits. Previous studies have shown that AMPK plays significant roles in cell proliferation, migration, and apoptosis. In addition, HG administration attenuates AMPK signaling activation, as reflected by the decreased AMPK phosphorylation (Wang et al., 2017; Zhu et al., 2017). Furthermore, metformin increases AMPK catalytic α subunit phosphorylation at Thr172, an important activation phosphorylation site that helps promote the pleiotropic effects of AMPK (Zhou et al., 2001). Metformin improves left ventricular function and survival via promoting AMPK and eNOS phosphorylation and increasing PGC1α expression in a murine model of heart failure (Gundewar et al., 2009). In addition, metformin inhibits tumor necrosis factor (TNF)-α-induced nuclear factor κB (NF-κB) activation via AMPK activation in HUVECs (Hattori et al., 2006). Furthermore, metformin induces anticancer effects dominantly via AMPK pathways (Ikhlas and Ahmad, 2017). These findings are in agreement with our *in vitro* data: the HG treatment decreased AMPK phosphorylation, while metformin specifically induced AMPK phosphorylation. Collectively, metformin improves HG-injured endothelial cell proliferation and reduces apoptosis by regulating BDNF expression in an AMPK-dependent manner (Figures 6, 7 and Supplementary Figures S1, S3). Metformin inhibits hepatic gluconeogenesis in an LKB1- and AMPK-independent manner via decreasing the hepatic energy state (Foretz et al., 2010). Therefore, to explore whether metformin influences HUVECs in other mechanisms aside from AMPK, further studies are required.

Activated AMPK modulates numerous downstream targets, such as acetyl-CoA carboxylase (ACC) and 3-hydroxy-3-methylglutaryl (HMG)-CoA reductase. However, CREB is also a phosphorylation target of AMPK, as evidenced by an experiment with HEK-293 cells transfected with a CREB-driven luciferase reporter (Thomson et al., 2008; Thornton et al., 2016). Our present study argues that AMPK activation by metformin increased CREB phosphorylation, whereas AMPK inhibition reduced pCREB and BDNF expression. In addition, CREB inhibition had no effect on pAMPK (Figure 6). These results indicate that CREB is a downstream target of AMPK. In addition, this study does not conflict with previous studies that indicate BDNF induces protective cardiovascular effects. However, we did not explore the downstream targets of BDNF in the current study. The specific molecular mechanisms by which BDNF improves endothelial function will be interesting to study in future work, and we also need *in vivo* evidence to support our findings.

Collectively, the present study has demonstrated, for the first time, that metformin improves cell proliferation and ameliorates cell apoptosis via the AMPK/CREB/BDNF pathway. These studies have identified a novel mechanism by which metformin activates protective pathways to reduce endothelial injury in vascular diseases of diabetes by modulating the expression of BDNF, a neurotrophic factor.

## Conclusion

We have reported evidence for a novel mechanism by which metformin protects the vasculature. We propose that metformin therapeutically improves cell proliferation and ameliorates cell apoptosis via the AMPK/CREB/BDNF pathway. Our study provides cellular insight into and further verifies the therapeutic uses of metformin in the realm of diabetic CVD therapy which might slow disease progression and improve prognosis.

## Author Contributions

HJ and NL designed the study. XH, BW, and YS cultured cells and carried out experiments. JH and XW performed Western blots and ELISA assays. WM and YZ did proliferation and apoptosis assays. RX, YS, and JH prepared the figures and performed statistical analysis. XH wrote the manuscript. All authors have read and approved the manuscript.

## Conflict of Interest Statement

The authors declare that the research was conducted in the absence of any commercial or financial relationships that could be construed as a potential conflict of interest.

## Supplementary Material

FIGURE S1 Metformin regulated CREB and BDNF expression via activation of AMPK. **(A)** Western blot analysis of AMPKα, pAMPKα, pCREB, CREB and BDNF expression in HUVECs treated with NG, HG (33.3 mmol/L), HG + MET (0.01 mmol/L), HG + MET + CC (10 μM), HG + MET + A-769662 (10 μM) and HG + MET + AICAR (0.5 mmol/L). **(B)** ELISA analysis of BDNF expression in HUVECs treated with NG, HG, HG + MET, HG + MET + CC, HG + MET + A-769662 and HG + MET + AICAR. **(C)** Western blot analysis of AMPKα, pAMPKα, CREB, pCREB and BDNF expression in HUVECs treated with NG + siNC, HG + siNC, HG + MET + siNC and HG + MET + siAMPK. **(D)** ELISA assay of BDNF expression in HUVECs treated with NG + siNC, HG + siNC, HG + MET + siNC and HG + MET + siAMPK. The results are expressed as the means ± SD. ^∗∗^*p* < 0.01; One-Way ANOVA.

FIGURE S2 Effects of HG on BDNF expression in endothelial cells. **(A)** Western blot analysis of BDNF expression in HUVECs treated with different concentrations of glucose (5.5, 15, 33.3, and 60 mmol/L) and mannitol (0, 9.5, 27.8, and 54.5 mmol/L). **(B)** ELISA analysis of BDNF expression in HUVECs treated with different concentrations of glucose (5.5, 15, 33.3, and 60 mmol/L) and mannitol (0, 9.5, 27.8, and 54.5 mmol/L). The results are expressed as the means ± SD. ^∗∗^*p* < 0.01; One-Way ANOVA.

FIGURE S3 Compound C reversed the effects of metformin on activation of AMPK in HG-injured endothelial cells. Western blot analysis of AMPKα and pAMPKα expression in HUVECs treated with NG, HG (33.3 mmol/L), HG + MET (0.01 mmol/L) and HG + MET + CC (10 μM).
