# Supplementary figures and images for: Metformin Modulates High Glucose-Incubated Human Umbilical Vein Endothelial Cells Proliferation and Apoptosis Through AMPK/CREB/BDNF Pathway
Source: Front Pharmacol. 2018 Nov 6;9:1266. doi: 10.3389/fphar.2018.01266 (PMC6232387; doi:10.3389/fphar.2018.01266)

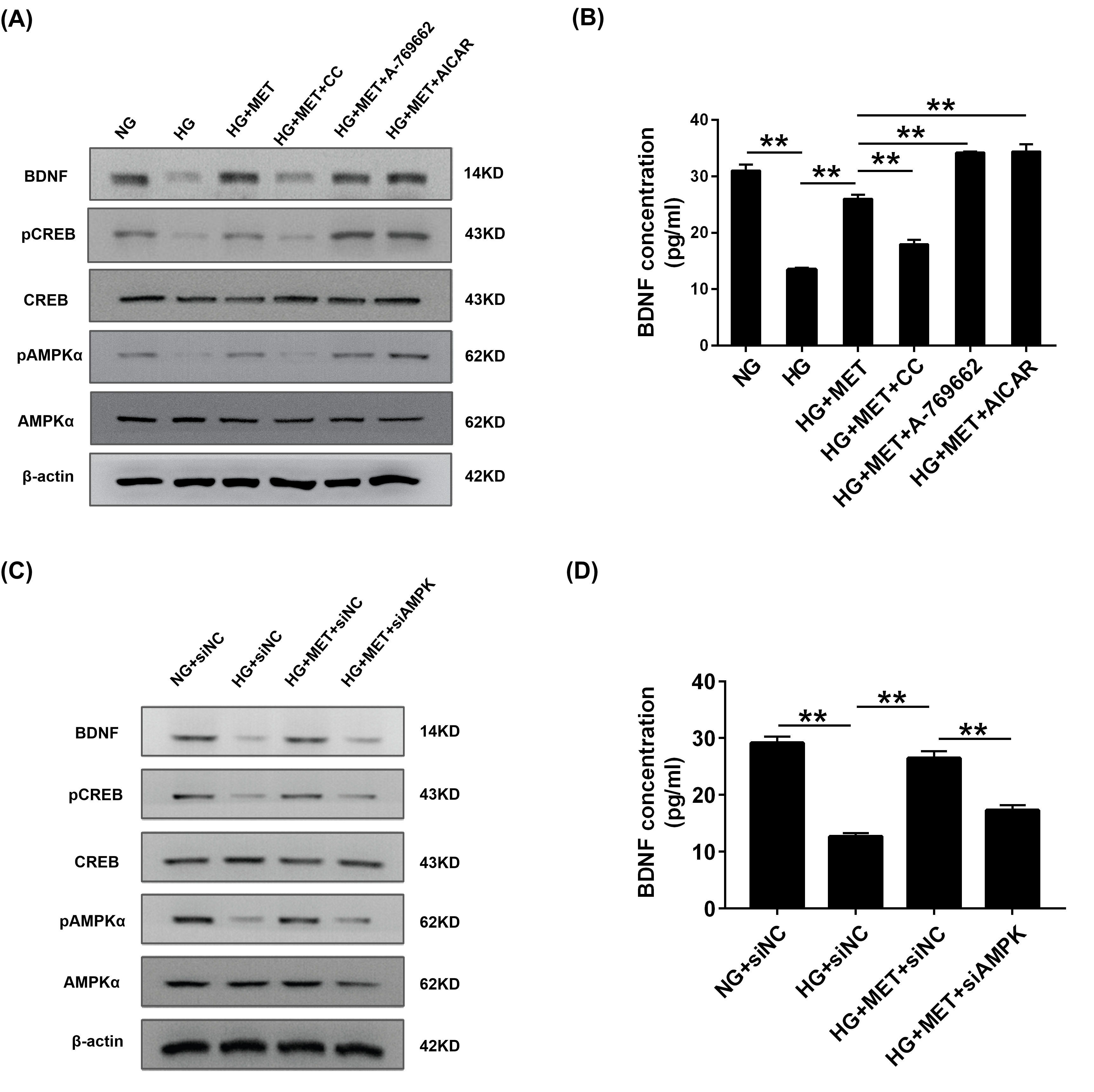

Supplement: FIGURE S1 — Metformin regulated CREB and BDNF expression via activation of AMPK. (A) Western blot analysis of AMPKα, pAMPKα, pCREB, CREB and BDNF expression in HUVECs treated with NG, HG (33.3 mmol/L), HG + MET (0.01 mmol/L), HG + MET + CC (10 μM), HG + MET + A-769662 (10 μM) and HG + MET + AICAR (0.5 mmol/L). (B) ELISA analysis of BDNF expression in HUVECs treated with NG, HG, HG + MET, HG + MET + CC, HG + MET + A-769662 and HG + MET + AICAR. (C) Western blot analysis of AMPKα, pAMPKα, CREB, pCREB and BDNF expression in HUVECs treated with NG + siNC, HG + siNC, HG + MET + siNC and HG + MET + siAMPK. (D) ELISA assay of BDNF expression in HUVECs treated with NG + siNC, HG + siNC, HG + MET + siNC and HG + MET + siAMPK. The results are expressed as the means ± SD. ∗∗p < 0.01; One-Way ANOVA. [file Image_1.TIF]

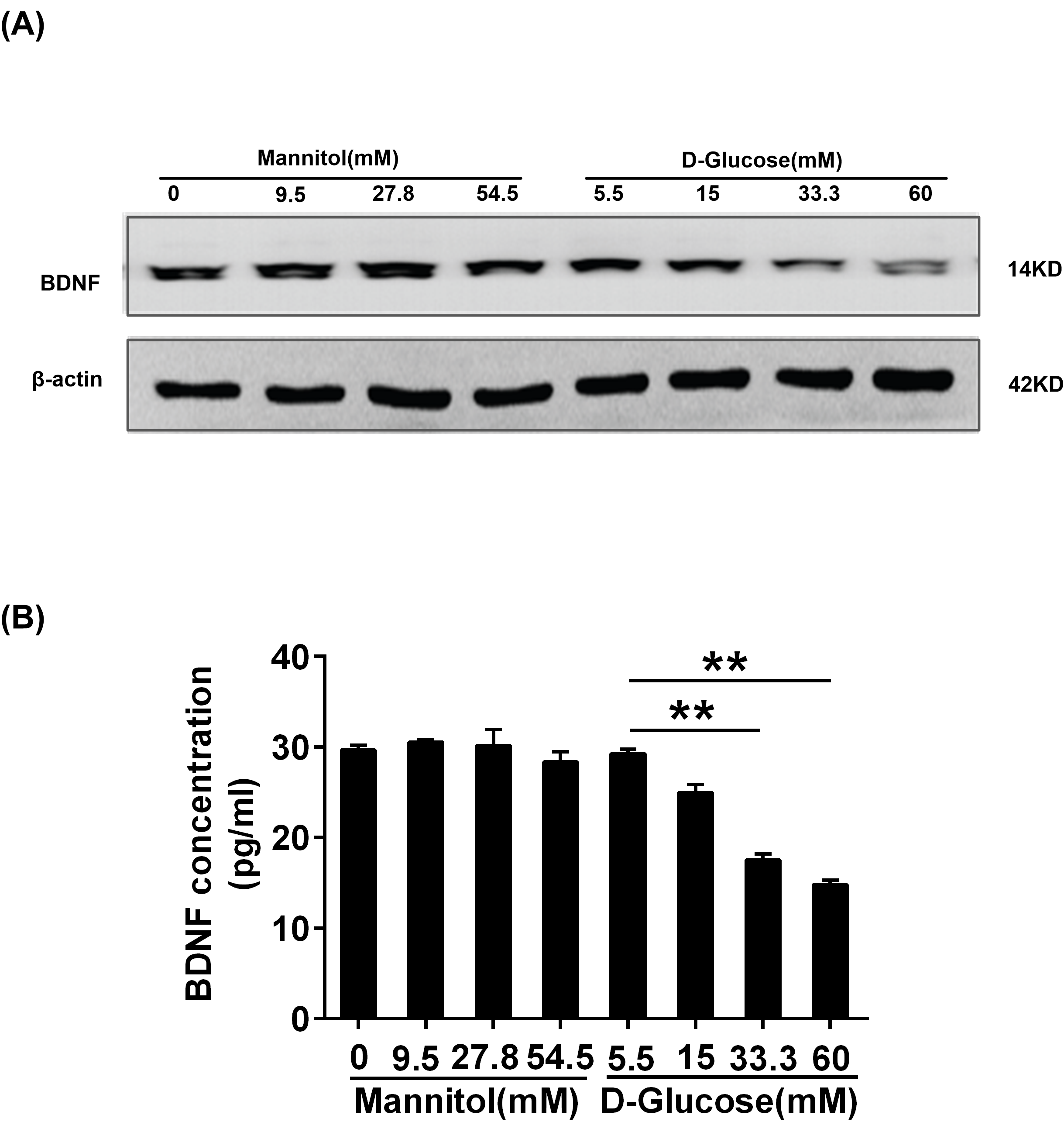

Supplement: FIGURE S2 — Effects of HG on BDNF expression in endothelial cells. (A) Western blot analysis of BDNF expression in HUVECs treated with different concentrations of glucose (5.5, 15, 33.3, and 60 mmol/L) and mannitol (0, 9.5, 27.8, and 54.5 mmol/L). (B) ELISA analysis of BDNF expression in HUVECs treated with different concentrations of glucose (5.5, 15, 33.3, and 60 mmol/L) and mannitol (0, 9.5, 27.8, and 54.5 mmol/L). The results are expressed as the means ± SD. ∗∗p < 0.01; One-Way ANOVA. [file Image_2.TIF]

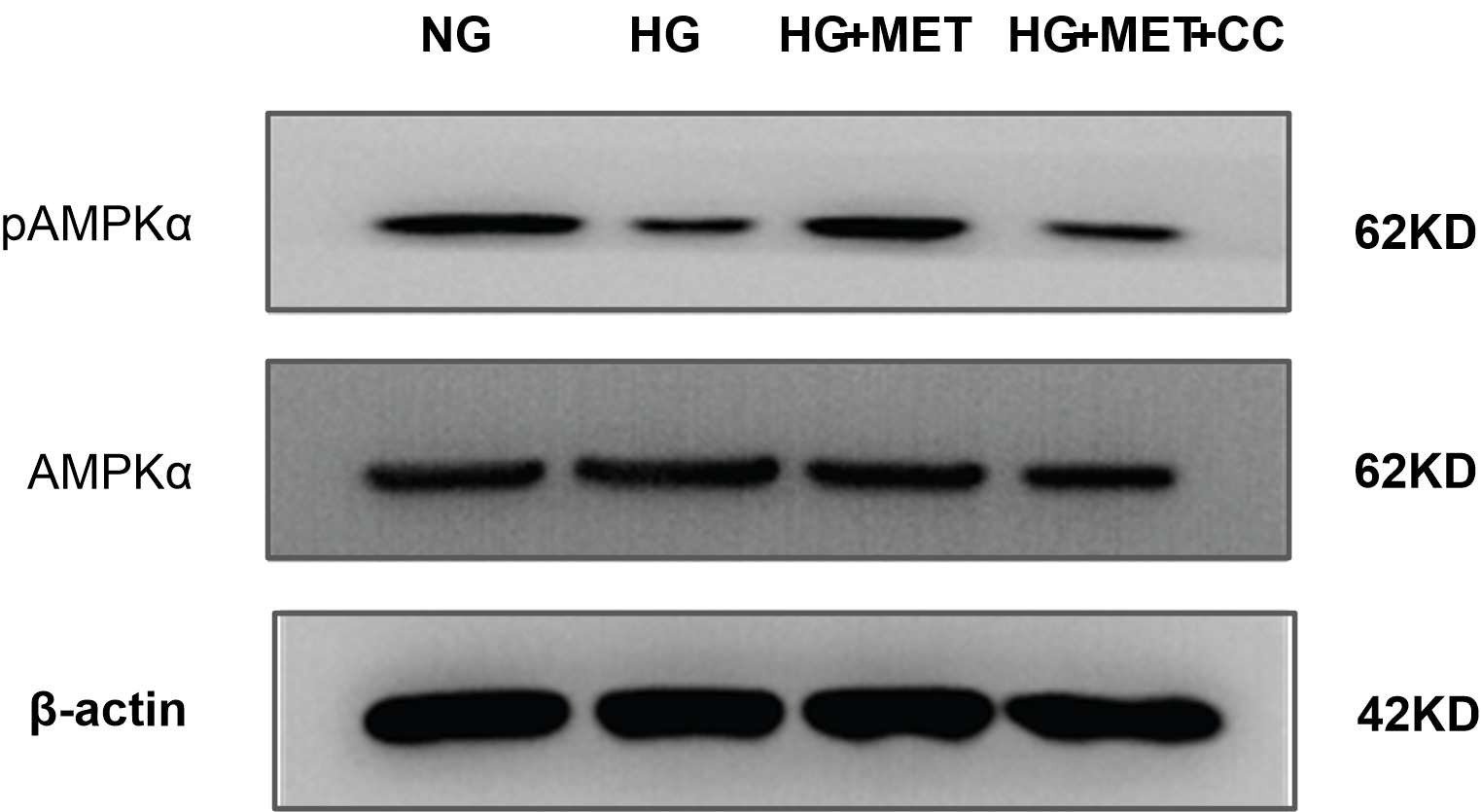

Supplement: FIGURE S3 — Compound C reversed the effects of metformin on activation of AMPK in HG-injured endothelial cells. Western blot analysis of AMPKα and pAMPKα expression in HUVECs treated with NG, HG (33.3 mmol/L), HG + MET (0.01 mmol/L) and HG + MET + CC (10 μM). [file Image_3.TIF]
